# Technological innovation for workload allocation in nursing care management: an integrative review

**DOI:** 10.12688/f1000research.125421.3

**Published:** 2024-04-26

**Authors:** Maria Alejandra Galiano, Maria Elisa Moreno Fergusson, William J. Guerrero, Maria Francisca Muñóz, Germán A. Ortiz Basto, Juan Sebastián Cardenas Ramírez, Maryory Guevara Lozano, Ana Larraín Sundt

**Affiliations:** 1 Clínica Universidad de los Andes, Santiago de Chile, Chile; 2 Faculty of Nursing and Rehabilitation, Universidad de La Sabana, Chía, Colombia; 3 Faculty of Engineering, Universidad de La Sabana, Chía, Colombia; 4 Clínica Universidad de La Sabana, Chía, Colombia

**Keywords:** Health Information Management, Nursing Care Management, Workload, Personnel Staffing, Scheduling Information Systems

## Abstract

**Background:**

Technology reduces the nursing workload, improve the quality care processes, patient’s safety, and avoid staff burnout. Innovative technologies are disrupting healthcare systems by improving the efficiency of processes and management. There is a discussion on the benefits, challenges, and barriers of these technologies and considering human factors of nursing management. The aim was to analyze the influence of technologies on the distribution of workload for nursing care management.

**Methods:**

An integrative literature review was performed. Four databases were searched: Scopus, Scielo, PUBMED, and CINALH following PRISMA guidelines. Articles published from January 2016 to December 2020, published in English, Spanish and Portuguese were included. Studies were excluded when they were not original research, did not met the quality criteria or they did not answer the research questions. Quality appraisal was performed using the Crowe Critical Appraisal Tool version 1.4 (CCAT). Two reviewers independently examined the title and abstract for eligibility according to the inclusion and exclusion criteria.

**Results:**

2818 potentially relevant articles were found, but once the inclusion and exclusion criteria in the abstracts were analyzed, 177 remained for evaluation. After following the PRISMA Guidelines, 35 studies were included in the review. Three categories were identified: Nursing workload; Information technologies and technological means for management; Technology acceptance.

**Conclusions:**

Technology has the potential to improve care management by estimating nurse workload in ICUs and non-critical units, but scientific evidence is more detailed in the former type of services. The literature provides insights about the factors that factors and the barriers that promote the technology acceptance and usability. We did not find studies comparing technologies and no scientific evidence proving improvements in care.

## Introduction

The nursing staff contribution is essential to guarantee quality and access to health services, as well as the outcomes of care for patients and their families. New technologies should support nursing management and decrease workload to meet the needs of the patients. This aspect is relevant since nursing care is essential for the patient recovery and safety ( Moreno-Monsiváis *et al*. 2015).

According to the World Health Organization, there is a relationship between the nursing workload, the morbidity, and mortality of hospitalized patients ( Ball *et al*., 2018) and after discharged ( Kim *et al*., 2020). Nevertheless, the evidence is not strong to determine the best nurse-to-patient ratio and the effects on the nursing workload and patient outcomes ( Coster *et al*., 2018).

The nursing workload is the amount of time, physical and cognitive effort needed to provide nursing care, in addition to activities related to service management and professional development ( Pedroso *et al*., 2020). Excessive nursing workload goes against the values of humanization of care, patient outcomes, patient safety ( Arango *et al*., 2015), quality of care ( Romero-Massa *et al*., 2011; Chang *et al.*, 2019), omitted care ( Moreno-Monsiváis *et al*., 2015) and nurses’ health ( Harvey *et al*., 2020). There is interest in creating technology to integrate scientific evidence and nursing expertise to reveal the relationship between nurse workload, burnout, and care quality ( Farid *et al*., 2020). Thus, an interdisciplinary research team, including nurses and engineers, working together is required to understand this relationship.

The aim was to analyze the use of technologies on the distribution of workload for nursing care management. The following research questions (RQ) were addressed:

• What methods are used to calculate nursing workload?
• What factors influence nursing care management technology acceptance by nursing professionals?

## Methods

An integrative literature review was performed using the Preferred Reporting Items for Systematic Reviews and Meta-Analyses (PRISMA) 2020 guidelines. Studies were retrieved from SCOPUS, Scielo, PUBMED, and CINAHL in January 2021. The search strategy was: (“analytics” OR “operations research” OR digital* OR “IT” OR technolog*) AND (“nursing workload” OR “nurse-patient ratio” OR “nursing workforce planning” OR “nursing algorithm” OR “nursing personnel staffing and scheduling” OR “nursing staff”)). The authors were involved in the identification, screening, and analysis stages. Inclusion criteria considered studies that address the integration of technology on nursing workload, burnout and nursing acceptance, published in full text in English, Spanish or Portuguese classified as scientific articles or reviews, in the areas of nursing, medicine, social sciences, engineering, published between January 2016 and December 2020. This date range was considered to focus on the recent technologies. Articles related with technology that didn’t analyze the impact on nursing workload or didn’t present technologies were excluded.

For all research, this must include a final section including details of ethical approval, informed consent and, where relevant registration.

**Figure 1.**
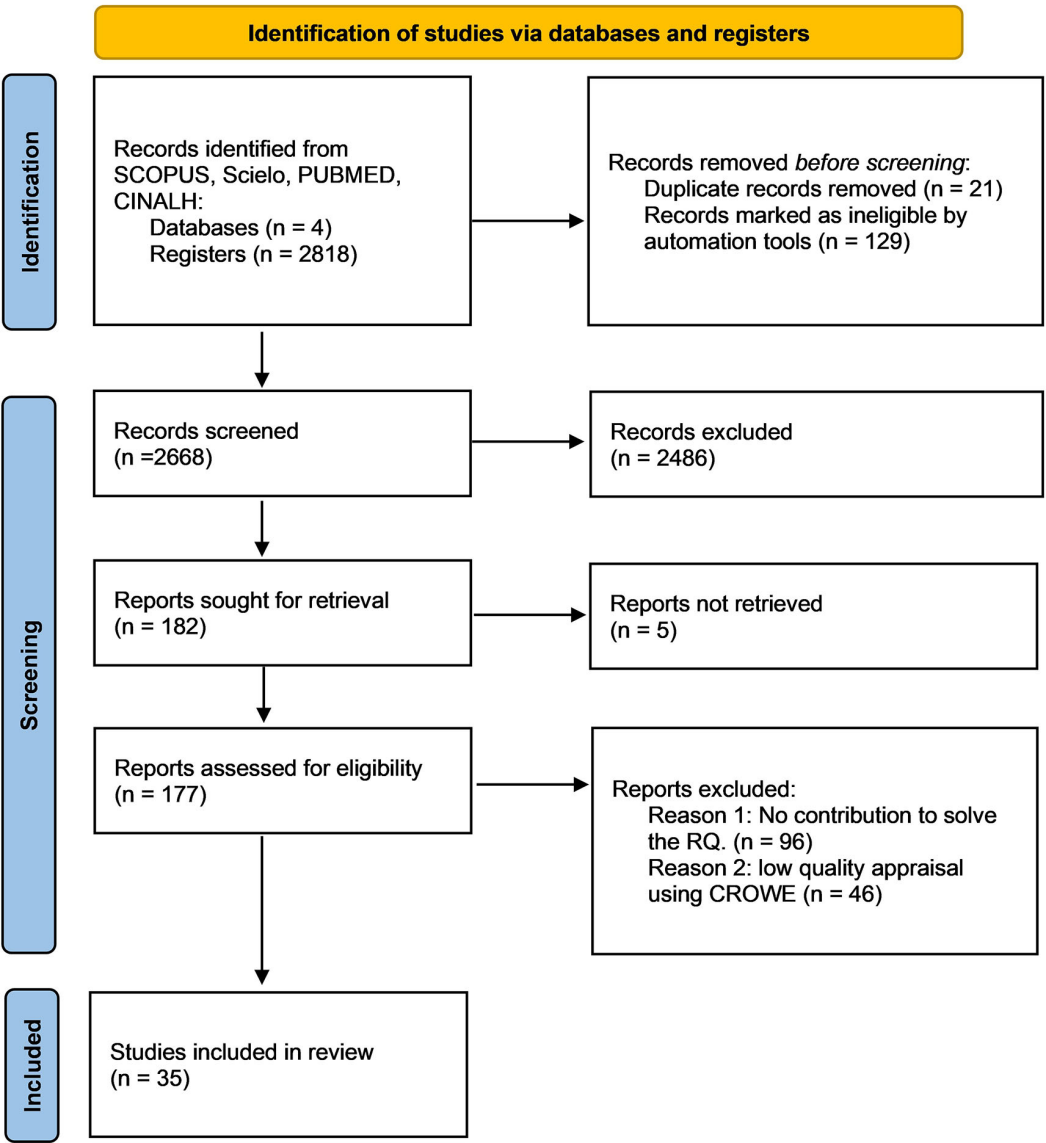
Flow chart of the study screening process.

Initially 2,818 articles were potentially relevant, of these 21 that were duplicates were eliminated and 129 were discarded by automation tools because they didn’t meet the inclusion criteria. In the second phase, the titles of 2668 articles were screened and 2486 were excluded since they didn’t meet the inclusion criteria. Later, the abstracts of 182 articles were analyzed. In the third phase, two independent reviewers read the full texts and discarded 96 articles because they didn’t contribute to the RQ and 46 because they obtained a low score in the CCAT. Ultimately, 35 studies were included ( Figure 1).

Quality review of each article was independently reviewed by two members of the research team/authors using the CCAT, that has shown to have high reliability to rate research papers ( Crowe *et al*., 2011). This tool is divided into eight categories and 22 items; each category receives a score on a 6-point scale from 0 (the lowest score) to 5. The total score is expressed as a percentage, and we arbitrarily considered acceptable a score above 60%. When there were differences in the quality assessment of an article, team consensus was achieved by comparing the arguments and evidence.

Data extraction was performed identifying the purpose, the design, participants and context of the study, the type of information collected, and the results. The interdisciplinary work between engineers and nurses made it possible to resolve doubts about technological and nursing concepts. For data synthesis, we performed an inductive content analysis. The information was collected in our database, and then grouped into categories that responded the RQ.

## Results

### Characteristics of the studies

35 studies from 18 countries were included, 18 observational studies, two quasi-experimental, five qualitative, three mixed methods, four literature reviews and three project evaluations. 57.1% were assessed as high quality, 42.9% as medium quality ( Table 1).

**Table 1.** Purpose, design, participants and context, data collection method and outcome of each of the 42 studies included in the review.

Nursing workload						
Study	Purpose	Design	Participants and context	Data collection methods	Outcomes	Quality (Crowe)
Redley *et al.* (2019) Australia	To describe the co-development, acceptability, and feasibility of a point-of-care App to promote uptake of best practice recommendations and consolidate nurses’ knowledge for managing symptoms of neurocognitive disorders.	Mixed method observation study	Convenience sample of nurses from general medicine inpatient wards at two hospital sites large Australian public health service. Observed during delivery of 80.5 hr of care to 38 patients; the App (n = 32 patients); and individual and focus group interviews with nurses (n = 25).	Semi-structured observation tool, interviews, focus groups. Observations were taken by trained observers; all were registered nurses with clinical experience and received instruction on using the semistructured observation tool. Prior to data capture, all observers had conducted concurrent observations to establish inter-rater reliability. The BRAIN-TRK App recorded de-identified data on the total number of patients for whom the app was used during Stages 2 and 3, and the frequency the App was used. Data for 32 unique patients were extracted from the App across the two wards. NUMs’ and ward nurses’ perceptions about the app were elicited throughout the workshops, meetings and testing, and eight individual and five focus group (n = 17) interviews.	The App included three components: cognition and risk assessment; tailored evidence-based strategies; and monitoring and evaluation of effectiveness. Observation data captured nurses using the App with 44.7% (n = 17) of eligible inpatients. Cognitive screening was completed at least once for each patient, with 146 risk assessments recorded. Interview data indicated the App’s acceptability was enhanced by familiarity and perceived benefits, but hindered by perceived increases in workload, inconsistent use, pressure to use the App and resistance to change. Feasibility and usability were enhanced by easy navigation, and clear and useful content, but hindered by unclear expectations, unfamiliarity and device-related factors.	Excellent
Griffiths *et al.* (2020) United Kingdom	To determine whether the Safer Nursing Care Tool corresponds to professional judgement, to assess a range of options for using the Safer Nursing Care Tool and to model the costs and consequences of various ward staffing policies based on Safer Nursing Care Tool acuity/dependency measure.	Observational study	81 medical/surgical wards in four NHS hospital trusts.	Daily measurements of the SFC tool, Hospital administrative systems, staff reports and national reference costs.	When there are fewer staff than the tool suggests are needed, the chance of nurses reporting ‘enough staff for quality’ is lower. However, other factors not considered in the tool (ward type, single rooms, day of the week, time of day) also affect reported staffing adequacy. Employing fewer nursing staff can be cheaper but often there are not enough staff on the wards because temporary staff cannot make up the shortfall. This increases the risk of death and longer hospital stays for patients. Although it is more expensive, we found that employing more staff, at the level needed to meet the demand observed on 90% of days, could provide value for money because outcomes were improved at modest cost.	Excellent
Simonetti *et al.* (2020) Chile	To measure and analyze associations between nurse organizational factors, such as staffing ratios and skill mix, and job outcomes in public hospitals in Chile.	Observational, cross-sectional	1,855 registered nurses working in medical-surgical units in 37 public hospitals	Data collection followed the RN4CAST research protocol. - Structured survey - Validated subscale to the Spanish of Emotional Wear of Maslach Burnout Inventory	The average staffing ratio was 14 patients-per-nurse, and the average skill mix was 31% registered nurses. Of all nurses, 35% reported burnout, 22% were dissatisfied, and 33% intended to leave. Being burned out increased by 9 and 6% the odds of being dissatisfied and the intent to leave, respectively (Odds ratio (OR) 1.09, p < 0.01 and 1.06, p < 0.01). Being dissatisfied increased by five times the odds of intent to leave (OR 5.19, p < 0.01).	Excellent
Tuominen *et al.* (2020) Finlandia	To identify nurse managers’ daily tasks during the rescheduling of sudden nursing staff absences by comparing two techniques: a paper-based system as phone calls and emails or information technology-based staffing systems. In addition, it is intended to evaluate the usability of information technology-based staffing solutions and evaluate estimated cost savings by using hospital permanent staff to cover sudden absences.	Quasi-experimental study design of a pre and post test group	nurse managers’ (n = 61) daily tasks (n = 5800) during rescheduling nursing staff sudden absences (n = 2628)	Observations and estimates of cost savings generated by the proposed intervention.	The number of nurse manager tasks during rescheduling decreased significantly (P < .001) as well as unstaffed shifts (P < .001) and unplanned shift changes (P < .001) after the information technology– based scheduling system was implemented. The usability score ranged from 76 to 100.	Excellent
Youn, Seok Hwa *et al.* (2020) South Korea	To compare nursing workload characteristics between the TICU and non-TICUs.	Retrospective observational study	One trauma intensive care units (TICUs) and five non-TICU where analyzed from September 2014 to August 2015 at a tertiary referral trauma center in South Korea. A total of 332 trauma patients in the TICU and 2,346 nontrauma patients in non-TICUs were studied.	“5-day observations and a survey with 18 questions about the impacts of ADCs on patient safety, a question about overall satisfaction and two open-ended questions about suggestions for improvements and free comments.”	Nurses were satisfied with ADCs with positive attitudes in the OR and ICU. This technology made their work easier, reduced the time and errors and more time was spent on direct patient care activities. Some resistance to change was observed in the OR.	Some limitations
Hope *et al.* (2019) United Kingdom	To explore the impact of using electronic data in performance management to improve nursing compliance with a protocol	Qualitative interpretative study	17 acute hospital nursing staff, General hospital in the South of England.	Semi-structured interviews were done by the main author face to face. The interviews were taped and transcribed verbatim. Data saturation was achieved with 16 interviews. Subsequently, the main author and the fourth author coded the transcriptions. The codes and the codification were discussed through NVivo memorandums and periodic meetings until a consensus was reached.	Introducing automated electronic systems to support nursing tasks decreases nursing burden but removes invisible mechanisms for negotiation that provide a balance between nursing judgement and standardized protocols. This can result in covert resistance that decreases patient safety.	Excellent
Assaye *et al.* (2020) Australia	To determine the effect of nurse staffing on patient and nurse workforce outcomes in acute care settings within low- and middle-income countries	A quantitative systematic review	27 studies were included. Empirical studies that addressed acute care nurse staffing levels, such as nurse-to-patient ratio or nurses’ qualifications, experience, and skill mix, and their influence on patient and nurse workforce outcomes were included in the review.	Studies published until July 2019 were identified from CINAHL, PubMed, Scopus, Embase, PsycINFO,Cochrane Library, Web of Science, and ProQuest. The JBI approach to critical appraisal, study selection, data extraction, and data synthesis was used for this review. The level of evidence was determined using GRADEpro.	The level of evidence in the studies was low. Low nurse-to-patient ratio or high nurse workload was associated with higher rates of: in-hospital mortality, hospital-acquired infection, medication errors, falls, and abandonment of treatment. Findings on the effect of nurse staffing on length of hospital stay and incidence of pressure ulcers were inconsistent. Extended work hours, less experience, and working night or weekend shifts all significantly increased medication errors. Higher nurse workload was linked to higher levels of nurses’ burnout, needlestick and sharps injuries, intent to leave, and absenteeism.	Excellent
Dall’Ora *et al.* (2020) United Kingdom	To provide a comprehensive summary of research that examines theorized relationships between burnout and other variables, in order to determine what is known (and not known) about the causes and consequences of burnout in nursing, and how this relates to theories of burnout	A theoretical review	91 papers were identified. The majority (n = 87) were cross-sectional studies	This theoretical review was conducted according to the methodology outlined by Campbell *et al.* and Pare *et al.* it was considered appropriate to use PRISMA-ScR as a guide. The search comprises articles published from 1975 to 2019, using MEDLINE, CINAHL, and PsycINFO.	The patterns identified by these studies consistently show that adverse job characteristics-high workload, low staffing levels, long shifts, and low control-are associated with burnout in nursing. The potential consequences for staff and patients are severe. The literature on burnout in nursing partly supports Maslach’s theory, but some areas are insufficiently tested, in particular, the association between burnout and turnover, and relationships were found for some MBI dimensions only.	Excellent
Aiken *et al.* (2021) Chile	To determine the associations of hospital nursing staff and work environments in Chile with the clinical results of patients and productivity.	Observational, cross-sectional	45 hospitals, 40 (88%) agreed to participate. Of the 2173 nurses from adult medical-surgical units invited to participate, 1652 nurses responded (76%). A total of 2,013 patients were recruited and data from 761,948 discharged medical-surgical adult patients were recorded.	The nurse work environment was measured with the internationally validated Practice Environment Scale of the Nursing Work Index (PES-NWI. The outcome variables from patient diagnosis-related groups (DRGs) data included mortality, readmission, and length of stay. Survey data were collected from patients in the same hospitals, using a five-page instrument with 22 items from the Hospital Consumer Assessment of Healthcare Provider and Systems patient experience and satisfaction survey, translated and validated in Spanish. Additional measures were used to control for potentially confounding factors.	Nurse workloads across public hospitals vary substantially, from nine to 24 patients per nurse, a remarkable difference in a public hospital system. Every additional patient added to the average nurse’s workload increased patients’ risk of in-hospital death by 4%. Patients in hospitals with 18 patients per nurse, compared with those in hospitals with eight patients per nurse, had 41% higher risk of death, were 20% more likely to be readmitted within 30 days of discharge, had stays that were 41% longer, and were 68% less likely to rate the hospital highly and 55% less likely to recommend the hospital to family and friends.	Excellent
Magalhães *et al.* (2017) Brazil	To describe the workload of the nursing team and demonstrate its association with patient safety outcomes in clinical and surgical inpatient units of a university hospital.	Observational, cross-sectional	Patients and professionals working in the 11 clinical and surgical units of the institution. 157,481 patients, 502 nursing professionals and 264 observations of safety outcomes.	The calculation of the workload of the nursing team was expressed as the ratio between the mean number of patients and the mean number of professionals working in the 24 hours (M+A+N) and for the day shifts, considering only the sum of professionals in the morning and afternoon shifts (M+A), divided into the categories nurse and nursing technician. For the calculation of the ratio of patient per nurse and nursing technician, the number of nursing professionals working from Monday to Friday was considered, excluding holidays and weekends. Patient data were collected through the institution’s Management Information System	The ratios of patients per nurse and per nursing technician in day shifts indicate a mean estimate of 14-15 and 5-6 patients per professional, respectively. There was a significant association between the workloads in the inpatient units and average length of stay, urinary infection related to invasive procedure and the satisfaction of patients with nursing care.	Some limitations
Harris *et al.* (2018) USA	Characterize Health Information Technology (HIT) use and measure associations between Electronic Health Record (EHR)-related stress and burnout among Advanced Practice Registered Nurses (APRNs).	Observational, cross-sectional	371 APRNs licensed and in practice in Rhode Island	Electronic survey. The survey period was from May 8th, 2017 to June 12th, 2017. Burnout was measured using a single question item from the Mini z (a 10-item survey developed from the Physician Work Life Study). Three EHR-related stress measures were adopted from the Mini z	Of the 371 participants, 73 (19.8%) reported at least one symptom of burnout. Among participants with an EHR (N = 333), 165 (50.3%) agreed or strongly agreed that EHR added to their daily frustration and 97 (32.8%) reported an insufficient amount of time for documentation. After adjustment, insufficient time for documentation (AOR = 3.72 (1.78–7.80)) and EHR adding to daily frustration (AOR = 2.17 (1.02–4.65))) continued to be predictors of exhaustion.	Excellent
Pekince and Aslan (2020) Turkey	To determine the work-related strain levels of the nurses working in a university hospital and the influencing factors.	Descriptive study	445 Nurses working at Fyrat University	"Two data collection forms were used: The personal data form prepared by the researcher to determine the sociodemographic and work conditions of nurses and the Work Related Strain Inventory (WRSI). The data was collected by performing the face-to-face interview technique."	It was determined that marital status, choosing the profession willingly, and job satisfaction were the variables that were effective on the work-related strain level (p<0.05).	Excellent
De Oliveira, Garcia, Nogueira (2016) Brazil	To identify evidence of the influence of nursing workload on the occurrence of adverse events (AE) in adult patients admitted to the intensive care unit (ICU).	Systematic review	Eight studies comprised the final sample of the review	The review was conducted in the databases MEDLINE, CINAHL, LILACS, SciELO, BDENF, and Cochrane from studies in English, Portuguese, or Spanish, published by 2015. The analyzed AE were infection, pressure ulcer (PU), patient falls, and medication errors.	Six studies (75.0%) identified the influence of work overload in events of infection, PU, and medication errors. The Nursing Activities Score (NAS; 37.5%) and the Therapeutic Intervention Scoring System (TISS; 37.5%) were the instruments most frequently used for assessing nursing workload.	Excellent
Kang, Kim, Lee (2016) Korea	To investigate the correlation between nursing workload and nurse-perceived patient adverse events.	Observational, cross-sectional	1,816 nurses working in general inpatient units of 23 tertiary general hospitals in South Korea.	Survey methods. Nurses answered the question: “How often did you perceive in-hospital accidents and patient side effects over the past year?” as dependent variable. The explanatory variable was the workload of the nurses, measured using the amount of the non-nursing tasks performed; the bed to nurse ratio, and the nurses’ subjective perception regarding whether or not sufficient workforce is available. Data is analyzed through multilevel logistic regression.	The study suggested that the high level of nursing workload in South Korea increases the possibility of patient adverse events.	Some limitations
Carlesi *et al.* (2017) Chile	To identify the relationship between the workload of the nursing team and the occurrence of patient safety incidents linked to nursing care in a public hospital in Chile.	Observational, cross-sectional	879 patients from 11 units of a public hospital, 85 nurses and 157 nursing assistants who were performing their duties in these units during the study period	The estimation of workload in Intensive Care Units (ICUs) was performed using the Therapeutic Interventions Scoring System (TISS-28) and for the other services, we used the nurse/patient and nursing assistant/patient ratios.	The overall incident rate was 71.1%. It was found a high positive correlation between variables workload and rate of falls. The medication error rates, mechanical containment incidents and self-removal of invasive devices were not correlated with the workload.	Excellent
**Information technologies and technological means for management**						
Sato *et al.* (2016) Japón	To utilize data from the developed ward management tool to consider workflow processes for nursing staff and the relationships between the nursing competence of the nursing staff and the patients’ conditions and how these impact on workloads.	Evaluation of a management tool to support efficient management of ward-based nursing tasks using ICT, visualization of workload and active use of data.	68 nurses of surgical and circulatory organ medical ward	Survey methods. Using the NCAM system, the Nursing staff of the target ward entered the actual information, task completion time at the required time, and also the planned completion time. Data from the ward management tool was used to consider workflow processes for nursing staff and the relationships between nursing competency of nursing staff and patient conditions and how these impact workloads.	Workload trends showed a high degree of conformity to a model which accounted the influences of staff teamwork and patient condition. This suggested that the condition of the patient had a significant effect on the workload and, at the same time, the workload tended to decrease as the degree of teamwork increased.	Some limitations
Vorakulpipat *et al.* (2019) Thailand	Provide a design of the EasyHos system and the case study in hospitals in Thailand.	Quasi-experimental study	2 hospitals in Thailand, one small and the other with a medium size. During the operation trial, 10 on-duty nurses performed the experiment. The equipment was used to provide information to patients who did not want to install the app or did not have a mobile phone The operation trial was performed for a 30-day period in a real hospital situation	7 structured questions to obtain information about pretest and postest conditions. 3 categories of information were assessed: (1) the statistics on queuing queries and related subjects, (2) information regarding what the patients wanted to know and related subjects, and (3) business process features.	The questions from patients were reduced by 83.3% after using EasyHos system, nurses and hospital staff had 5 min more to do their routine work each day, (3) the patients rarely asked the nurses/hospital staff about their place in line and status, (4) the patients knew what to do and where to go from the information provided by the EasyHos system, (5) patients could go other places or do other things and relax while waiting on their doctor, (6) patients knew when to come back to the waiting area because EasyHos notified them and provided a map to assist in walking back to the waiting area, and (7) EasyHos had a double-check system for the hidden flaws in the process.	Excellent
Yu *et al.* (2019) Taiwan	To investigate the use of barcode technology as a method to improve the accuracy of pathology specimen labeling and patient safety. To assess nurses’ perceptions of system quality, information quality, service quality, user satisfaction, and net benefits.	Observational, cross-sectional study	68 perioperative nurses working at a 1000 bed university hospital in Taiwan	Survey through an instant messaging application and report of rejected pathology specimen data from the Pathology Department.	Nurses scored net benefits as highly contributing to their satisfaction, whereas system quality contributed most to dissatisfaction. Specimen management errors significantly decreased with barcode system implementation	Some limitations
Zhou *et al.* (2019) Ghana	To assess the “Social Influence” (SI) and “Facilitating Conditions” (FC) that support nurses’ acceptance of HEIMS using the “Unified Theory of Acceptance and Use of Technology” (UTAUT) model.	Observational, cross-sectional survey	660 nurses from 5 public hospitals that use HEIMS in Ghana.	Electronic platform questionnaire on smartphones. The UTAUT tool is used to characterize the social influence and facilitating conditions that support nurses’ adoption of hospital electronic information management system. AMOS Structural Equation Modelling (SEM) v.22.0 was employed.	Behavioral Intention” (BI) to HEIMS use was significantly predicted by Social influence (SI) and facilitating condition (FC) (p < 0.001). Notably, both SI and FC had an influence on nurses’ use behavior (UB) with behavioral intention (BI) as the mediator, which explains a total of 42.1% variance in the intention of nurses to use HEIMS. Likewise, UB of HEIMS was also significantly predicted by SI (R2 = 43.2) and BI (R2 = 0.39.6) with both constructs explaining a total of 51.7% of the variance in nurses’ acceptance to use HEIMS.	Excellent
Giraldo *et al.* (2018) Argentina	To know the perceptions and expectations of nurses regarding the implementation of BCMA (Bar Code Medication Administration)	Qualitative research with content analysis, objective description and a common perspective.	39 nurses from different inpatient wards of the Hospital Italiano de Buenos Aires, with and without implementation of the system.	A script composed with previously elaborated questions was applied to 5 operative groups.	Determinants for the acceptance of the system by the nursing staff: the ease of use of the mobile station, the device, the nursing application and its usefulness, and high expectations about the new process. Lack of reliability and interruptions impeded its use.	Some limitations
Leary *et al.* (2016) England	Determine if relationships between registered and non-registered nurse staffing levels and clinical outcomes could be discovered through the mining of routinely collected clinical data. Examine the feasibility and develop the use of ‘big data’ techniques commonly used in industry for this area of healthcare and examine future uses.	Observational, Data mining methodology.	A National Health Service (NHS) hospital in the Midlands (The Trust), which has 1,189 beds in 33 wards in 2 hospitals and a variety of care settings, such as acute medical care and surgical and specialized rooms.	Routinely collected physiological, signs and symptom data from a clinical database were extracted, imported, and mined alongside a bespoke staffing and outcomes database. The physiological data consisted of 120 million patient entries over 6 years, the bespoke database consisted of 9 years of daily data on staffing levels and safety factors such as falls. The staffing and key performance indicator (KPI) database was designed within the Trust in Excel and easily extracted.	The relationship between staffing and outcomes appears to exist. It appears to be non-linear but calculable and a data-driven model appears possible. After mining, 40 correlations (p<0.00005) emerged between safety factors, physiological data (such as the presence or absence of nausea) and staffing factors. Several inter-related factors demonstrated step changes where registered nurse availability appeared to relate to physiological parameters or outcomes such as falls and the management of symptoms.	Some limitations
Mau *et al.* (2019) USA	Determine if enhancements developed to support the use of an electronic Early Warning System (EWS) resulted in quicker stabilization of clinical deterioration.	Observational	Three medical-surgical units and a cardiac telemetry unit of the community hospital in Northeast Ohio. Data from all adult patients (18 years or older) admitted to the four study units during the study period (February 2014 to January 2016) and who did not have a “do not resuscitate, comforting care order” (DNR CC) were considered.	- Electronic medical record - Data on nurse educational makeup, turnover rate - A basic EWS scoring tool that uses the parameters (systolic blood pressure, pulse, respiratory rate and oxygen saturation) and weighting.	There was a significant (p < .001) decrease in time patients spent with higher risk EWS scores and time to reassessment following an EWS alert after implementing an enhanced EWS.	Some limitations
Farzandipour (2020) Irán	To evaluate the informatics competencies of nurses working in hospitals.	Cross-sectional study	200 questionnaires was distributed among nurses, 197 were returned with 98.5% of items answered.	The tool used to collect data was the localized Staggers standard questionnaire.	60.6% of nurses have few capabilities regarding informatics competencies. Also, practicing nurses were more able in the area of informatics knowledge rather than the other two areas of basic computer skills and informatics skills.	Excellent
Moon (2019) Korea	To suggest the efficient model for nurse resource management that can estimate optimum nurse resources according to the nursing intensity of the hospitalized patients	The study was performed with four steps including collection and analysis of requirements, system design, system realization, and evaluation	Development processes: 78 nurses who cared 483 hospitalized patients in internal medicines, 543 hospitalized patients in surgical departments, and 96 patients in ICU in a national university hospital, Korea. Evaluation of systems: 44 subjects including 22 head nurses and 22 charge nurses.	The study was performed with four steps including collection and analysis of requirements, system design, system realization, and evaluation. The measurement tool used in the step of system evaluation was a modified version of Questionnaire for User Interaction Satisfaction (QUIS)	This system contributed to an enhancement of the working process speed, efficiency, and accuracy by simplifying the works which were the purposes of the nursing information system, which changes dynamically, to support decision making on the management of effective and flexible nurse resources.	Excellent
Respicio *et al.* (2018) Brazil	To determine the number of nurses and of nursing technicians to be assigned to the permanent pool of a hospital unit, complying with the demand for healthcare per shift and minimizing salary costs. Method relies on a new mathematical model.	He proposed methodology follows two phases. A spreadsheet-based tool was developed to embed the model and to allow simulating different scenarios and evaluating the impact of demand fluctuations, thus supporting decision-making on staff dimensioning.	5 units of the São José hospital, Porto Alegre, Brazil, are considered	For each unit, the data referring to the demand for one day were considered, and the following were compiled: number of hospitalized patients requiring minimal care, intermediate care, semi-intensive care and intensive care. The data are organized in three tables: Patients data, COFEN parameters, contains the model’s parameters and Personnel data. The method is applied using a Microsoft Excel spreadsheet.	The method allows the determining of staff level per shift and skill depending on the mix of patients’ illness severity. The model and the developed tool are easily customizable to integrate the work rules of different hospital contexts.	Some limitations.
Massarweh, Tidyman, Luu (2017) USA	To provide a level of safety when performing the task of nurse-to-patient assignment by introducing a technology-enhanced tool – the eAssignment sheet.	Quality improvement project	"21 nurse managers multi-hospital system in Northern California that provides care for approximately 2,000 admitted patients across 108 distinct adult inpatient nursing units daily."	The eAssignment sheet was informed by nurse managers providing the workflow of nurse-to-patient pairing to an individual with expertise in Visual Basic® computer coding.	"The eAssignment sheet is one tool to assist the nurse manager in the critical and routine task of nurse to- patient pairing in a manner that promotes patient safety, equitable workload distribution, and may provide financial gains"	Some limitations.
**Technology acceptance**						
Beaney *et al.* (2019) England	To demonstrate that practical hands-on training with nurses committed to a digital future, is an effective way to create digital champions, who can implement TECS in general practice.	Qualitative descriptive	21 volunteer general practices nurses	Self-rated survey, phone survey and (LCAV) questionnaires (NHS England 2016)	The GPN seeded change in their practice teams; increased reported productivity, patient safety and compliance; and reduced avoidable practice face-to-face appointments and phone calls. Additionally, the results of the ALS programme provide evidence that initiatives adopted by just one member of a general practice team can evolve from a pilot to usual practice. The programme shows that a ‘ground-up’ approach to upskill and empower front-line clinicians is central to embedding technology-enabled care services (TECS).	Some limitations
Higgins *et al.* (2017) USA	To elucidate hospital nurses’ work activity through observations, nurses’ perceptions of time spent on tasks, and electronic health record (EHR) time stamps. Nurses’ attitudes toward technology and patients’ perceptions and satisfaction with nurses’ time at the bedside were also examined	Mixed-methods design and triangulated data capture approach that included prospective observations	79 nurses in 4 inpatient units of the University of Pittsburgh Medical Center (UPMC). 35 000 nurses activities and 215 patients	Observation and questionnaire carried out by authors. Observers used standardized logs for itemizing activity and recording nurses’ time spent performing specific tasks. To evaluate nurse characteristics and perceptions, a questionnaire with items about perceptions of the percentage of a work shift spent in direct patient care, indirect care, waste, and unit based/professional development activities. The questionnaire also asked about the perceptions of the time spent charting in the HER, two items assess the attitudes toward technology, and an open-ended question asking about factors that enhanced or interfered with their use of technology.	The findings demonstrate a discord between nurses’ perceptions of time spent in direct care, “waste” activities, and EHR charting compared with observations. In contrast, observed EHR activity converged with time stamps of activity generated by the Cerner Lights On Network technology. Although use of the EHR was the most time-consuming activity (followed by patient assessment and interaction, in-person communications with health care workers about patients or patient care, and patient care and bedside procedures), nurses reported positive attitudes toward technology, and patients’ indicated general satisfaction with the duration of direct nursing care time. The convenience and safety facilitated the nurses’ use of technology, whereas lack of reliability and interruptions impeded its use.	Some limitations
Tubaishat (2018) Jordan	To explore nurses’ perceptions of usefulness and ease-of-use of EHRs.	Observational, cross-sectional study	1539 nurses from 15 randomly selected hospitals, representative of different regions and healthcare sectors in Jordan.	Self-administered questionnaire, which was based on the Technology Acceptance Model	Jordanian nurses demonstrated a positive perception of the usefulness and ease-of-use of EHRs, and subsequently accepted the technology. Significant positive correlations were found between these two constructs. The variables that predict usefulness were the gender, professional rank, EHR experience, and computer skills of the nurses. The perceived ease-of-use was affected by nursing and EHR experience, and computers skill.	Excellent
Steege and Dykstra (2016) USA	To explore barriers and facilitators within the hospital nurse work system to coping and fatigue.	Qualitative study.	22 nurses (ICU and MSU) from a large academic hospital in the US.	Semi-structured interview guide based on the SEIPS model.	There were multiple themes characterizing barriers and facilitators to fatigue and coping within the hospital RN work system. Themes were identified in each of the five components of the SEIPS model. While some themes can be directly identified as contributing to or preventing fatigue, or as a barrier or facilitator to coping, the relationship between some of the themes and both fatigue and coping is more complex.	Some limitations
Tu *et al.* (2018) Taiwán	To examine staff perceptions of planned obsolescence during a transition from old to new handheld devices for long-term mobile healthcare.	Observational, cross-sectional study	120 nursing staff (aged 40 to 49) from 26 independent long-term care facilities, frequent computer users.	A questionnaire based on the modified technology acceptance model (TAM) was used. All participants attended two training sessions and received support for an expert in their institutions. Participants used the devices for 2 months in their institutions and 500 resident evaluations were done in total. SPSS (version 21.0; IBM SPSS Statistics, IBM Taiwan Corp, Taipei, Taiwan) was used for analysis. They used Cronbach’s α to determine the reliability of the TAM, descriptive and mean comparison statistics.	Although the new devices with advanced features scored significantly higher in acceptance ratings, users still expressed high acceptance of, satisfaction with, and willingness to use the old device, which featured an effective and carefully designed user interface.	Some limitations
Wood *et al.* (2019) Australia	To provide a broad overview of the range and variety of published research relating to medical/surgical nurses use of early warning score systems to detect deterioration amongst adult patients in the general ward settings.	Scoping review	23 articles were included in the review	Data is collected of the articles from 2008 to 2018. A five-step process was used in this scoping review including: identify the research question; search and identify the relevant studies; selecting relevant studies; charting the data; and collate, summarize and report the results. The PRISMA extension for scoping reviews was used to guide this scoping review. They assessed studies against the Cochrane Effective Practice and Organization of Care taxonomy of health service interventions (Effective Practice and Organization of Care (EPOC), 2016).	There are several factors that prevent nurses from using the early warning score system appropriately. Barriers include confidence and experience amongst nurses along with previous interactions with the rapid response team. Nurses state track and trigger charts are easy to use and help guide them on intervening when patients deteriorate, however they also state that the algorithms are difficult to follow due to workload and resource availability. There is also evidence indicating that nurses rely heavily on scores generated by track and trigger charts rather than patient assessment and despite the introduction of the worry criteria for rapid response team activation many clinicians are reluctant to use it. Nurses use early warning score systems to maintain safety for hospitalized patients. However, education sessions focusing on building nurse confidence and increased resources and staffing in busy times can help to improve the function of early warning score systems and patient safety further.	Excellent
Van der Veen *et al.* (2020) Netherlands	To identify potential risk factors associated with workarounds performed by nurses in Barcode-assisted Medication Administration in hospitals.	Prospective observational	5,793 drug administrations among 1,230 patients administered by 272 nurses in the units of: cardiology, lung diseases, geriatrics, internal medicine, neurology, surgery, and orthopedics of four hospitals in the Netherlands.	Disguised observation method Case report form. The observer accompanied the nurse who administering medication and observed the administration of each medication to the patient to identify potential errors and workarounds. The statistical analysis of data was made through logistic mixed models.	Potential risk factors associated with workarounds were the day of the week, the timing of the medication administration, the route of administration, the administration of medication from irregularly used ATC classes and the patient–nurse ratio. Other factors, such as the percentage of barcoded medication and work experience, were not associated with workarounds.	Some limitations
Ozan and Durgun (2020) Turkey	To determine nurses’ perceptions regarding the use of technology in nursing care practices.	Cross sectional study	A sample of 408 volunteer nurses that work in a university hospital in eastern Turkey from April to June 2017.	12 questions about nurses´ perceptions regarding the positive and negative effects of the use of technology.	“The nurses expressed positive perception about the use of technology in nursing-care practices. more female than male nurses thought that using technology makes nursing-care practices patient centered”	Excellent
Handayani *et al.* (2018) Indonesia	To identify and rank user acceptance factors regarding the implementation of hospital information systems (HIS) based on the views of the following user groups: doctors, nurses, hospital management, and administrative staff (operators).	Qualitative and quantitative design, using interpretive, descriptive and contextual strategies.	109 participants: doctors, nurses, administrators and hospital managers.	Qualitative data was collected through interviews and observation. Interviews were conducted with doctors and hospital management personnel. Interviews were recorded, transcribed, and coded to determine user acceptance factors regarding HIS. The most important HIS user acceptance factors were identified using quantitative data collected through questionnaires.	The study identified 15 important HIS user acceptance factors, which were ranked differently by each user group. The results show that non-technological dimensions, such as human and organizational dimensions, influence HIS user acceptance to a greater extent than technological dimensions	Excellent

### Elements of nursing management

**Figure 2.**
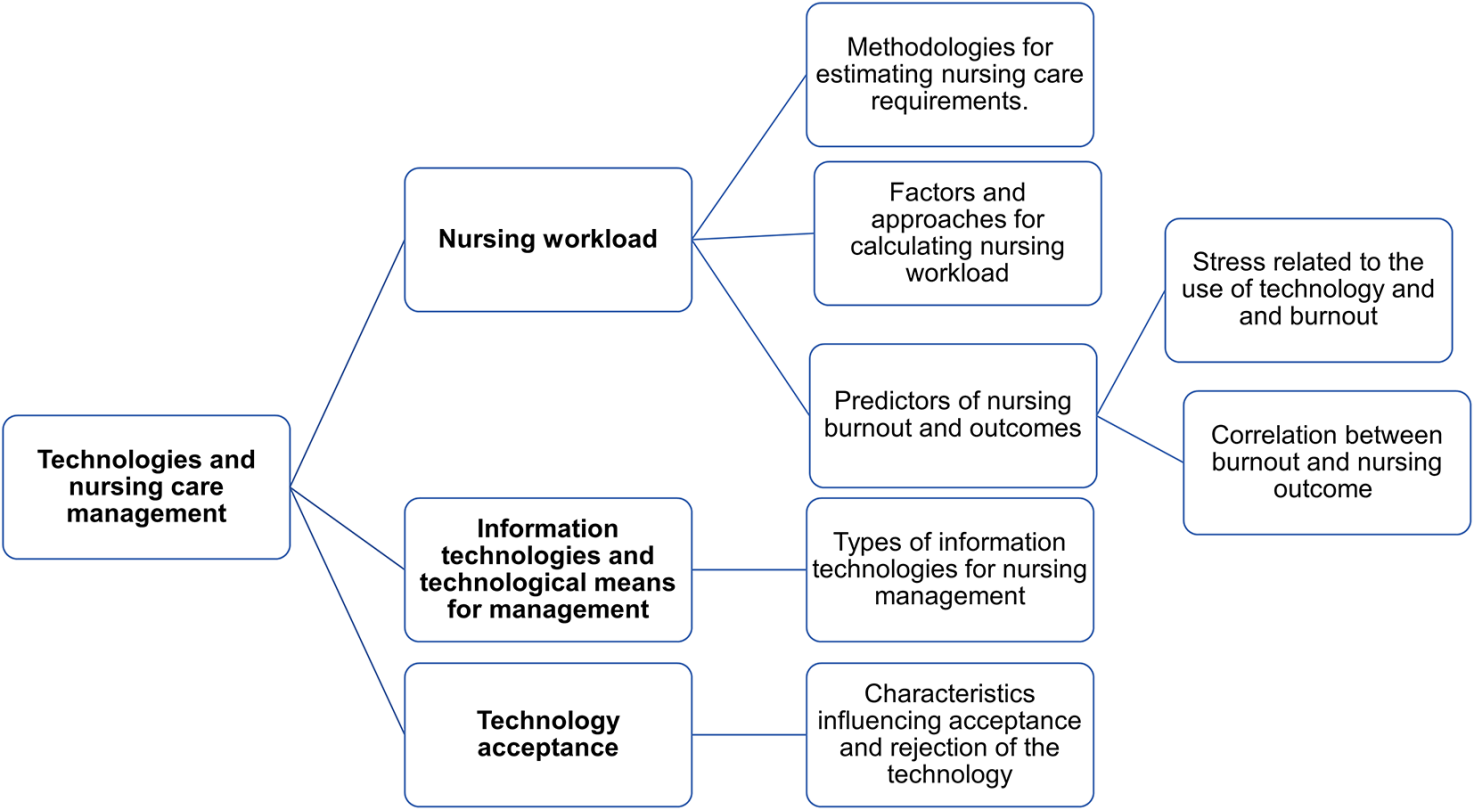
Concept map of the studies’ categories and results.

The studies were classified into three categories: 1. Nursing workload; 2. Information technologies and technological means for management; 3. Technology acceptance. Figure 2 presents a concept map of the categories discussed in the results.

- **Nursing workload**

This category includes the methodologies for estimating nursing care requirements, factors, and approaches for calculating nursing workload and the predictors of nursing burnout and outcomes.

The challenge for workload estimation and prediction systems is having adequate information about the time and effort needed for nursing care activities, considering the interactions and iterations of the day-to-day care process, in physical, cognitive, organizational, and emotional dimensions ( Redley *et al*., 2020). The variability in the patient care process usually has significant skewness. There are methodologies for estimating nursing care requirements, which are classified into those based on expert opinion, benchmarking, nurse-patient relationship, patient prototyping, multifactorial indicators, and task time-based approaches ( Griffiths *et al*., 2020).

There is literature about nurse-to-patient ratios, but the validity and reliability of the studies is weak ( Griffiths *et al*., 2020). In practice, legislation on minimum nurse staffing levels varies. In California (USA), law 394 of 1999 stipulates a 1:6 ratio. In Australia, the Safe Patient Care Act 2016 stipulates a 1:4 ratio. In Queensland, the daytime ratio is 1:4, and nighttime 1:7. In Wales (United Kingdom) the law stipulates a daytime ratio 1:5, and nighttime 1:7. Other countries, such as Colombia (South America), do not have legislation. The methodological proposals on how to calculate the nursing care requirements, nurse staffing, and skill mix, are based on local observations, which don’t allow generalization.

Low staffing levels negatively affect safety indicators and staff performance ( Simonetti *et al*., 2020), but estimating the nursing workload, considering emergent care, for decision making, is still an unsolved challenge ( Griffiths *et al*., 2020). There are no systems that estimate workload beyond *ad hoc* methodologies, based on average measurements that ignore the variability. The most currently used systems are:

• GRASP (Grace-Reynolds Application and Study of PETO) ( Clark & Poland, 1976).
• TOSS (Time Oriented Score System dedicated to intensive care patients) ( Italian Multicenter Group of ICU Research, 1991).
• NAS (Nursing Activities Score dedicated to intensive care patients) ( Miranda *et al*., 2003).
• The Safer Nursing Care Tool SNCT ( The Shelford Group, 2014).
• RAFAELA Patient Classification System including the Oulu Patient Classification instrument and the Professional Assessment of Optimal Nursing Care Intensity Level (PAONCIL) ( Aschan *et al*., 2009).
• Safecare by Allocate Software.

Comparisons of these systems show significant differences in the results ( Griffiths *et al*., 2020), and there is no evidence that one outperforms the others. In addition, the professional judgement-based approaches are popular and relatively reliable.

Commercial software computes staffing levels, coordinates nursing shifts, and plans nursing activities in case of unexpected staff absences. Tuominen *et al*. (2020) show that such tools reduce operating costs, the time required by nurse managers during the rescheduling process, the number of understaffed shifts, and unexpected changes in shift assignments.

The impact of technology for predicting nurse workload and deciding optimal staffing levels includes factors such as quality of care, patient safety, staff turnover and satisfaction, and process efficiency. Understanding nurses work is essential to assessing how technologies contribute to quality of care ( Redley *et al*., 2020). There has been insufficient discussion of how technologies can be used to correlate operational cost with quality of care ( Griffiths *et al*., 2020).

In relation to the predictors of nursing burnout and outcomes, patient and nursing staff outcomes are affected by nursing burnout and workload, which is significantly higher in ICUs than in other units ( Youn *et al*., 2020). Burnout is a problem that originates in individuals and organizations ( Maslach & Leiter, 2017). High nursing workload and poor skill mix are correlated with higher levels of burnout ( Assaye *et al*., 2020; Simonetti *et al*., 2020), as does a poor work environment ( Pekince & Aslan, 2020). In turn, burnout is associated with lower patient safety and omitted patient care ( Dall’Ora *et al*., 2020).

High nurse workload is associated with higher rates of mortality ( Assaye *et al*., 2020; Aiken *et al*., 2021), hospital-acquired infections, pressure ulcers, medication administration errors ( de Oliveira *et al*., 2016; Kang *et al*., 2016), falls ( Carlesi *et al*., 2017), treatment dropout ( Assaye *et al*., 2020), increased length of stay ( Magalhães *et al*., 2017) and readmissions ( Assaye *et al*., 2020; Aiken *et al*., 2021). It is also related to needlestick injuries, intent to leave, and absenteeism ( Assaye *et al*., 2020).

Harris *et al*. (2018) found a correlation between technology use and nursing burnout. The study showed that nurses experienced stress, burnout, and frustration with the use of EMRs and they reported insufficient amount of time for documentation.

The most studied technologies in ICUs (intensive care unit) were electronic medical records (EMR) and electronic medication management ( Redley *et al*., 2020). Nursing-specific information systems, general computer, communication technologies and electronic data analysis being less often studied. Time savings are proven with the use of technology, but the impact on clinical decision making, interpersonal relationships, and work stress that underpin nursing care quality is unproven with respect to direct benefits. These systems could fail to record legitimate reasons for missing data, which can reduce patient safety ( Hope *et al*., 2019).

- ***Information technologies and technological means for management***

Human and compassionate relationship between patients and nurses requires a meticulous synchronization between patients, healthcare staff, suppliers, processes, technologies, and information. The studies show that information technologies (IT) and technological means can support nursing care ( Vorakulpipat *et al*., 2019; Zhou *et al*., 2019).

IT include EMR and systems for: online data input, tracking patients, detect changes in the patient condition and workload reallocation, trace patient’s calls, hands-free communication between nurses, detecting falls, barcode technologies for medicine management, data mining on nursing flows, among others ( Massarweh *et al*., 2017; Respicio *et al*., 2018; Vorakulpipat *et al*., 2019; Yu *et al*., 2019; Zhou *et al*., 2019; Moon, 2019).

For effective IT implementation in nursing, change management and communication are determinants ( Giraldo *et al*., 2018) as well as technology skills training ( Farzandipour, 2020). It’s also recommended to follow workflows, multiplicity of task and patient care plans ( Vorakulpipat *et al*., 2019).

Current technology to assess the nursing workload doesn’t capture the complexity or quality of care, and doesn’t recognize the concurrent, iterative, and interrelated nature of cognitive processes and activities in nursing. Therefore, there is a gap in the literature on how technology affects the quality of nursing care and how to measure it ( Redley *et al*., 2020).

The patient’s condition has a significant effect on the workload ( Sato *et al*., 2016). The development of early warning systems for patient deterioration (systolic blood pressure, pulse, respiratory rate, and oxygen saturation), using analytics, shows a significant decrease in nursing care time ( Mau *et al*., 2019). These findings could be used to build a model for staffing ( Leary *et al*., 2016).

- ***Technology acceptance***

The factors that influence technology acceptance are the perceive usefulness, the ease-of-use ( Berg *et al*., 2017; Holden *et al.,* 2016), and the participation of nurses in its design ( Redley *et al*., 2019; Pendon *et al*., 2020). The human and organizational dimensions influence acceptance more than the technological dimensions ( Handayani *et al*., 2018). The use of technology is beneficial when it promotes productivity and nurses’ empowerment, patient safety ( Beaney *et al*., 2019), and quality of care ( Higgins *et al*., 2017). Also, there is a positive correlation between the perception of usefulness and ease-of-use of EMR, the scanner to record the medication administration, and the use of lifts to mobilize patients and nurses’ technology acceptance ( Tubaishat, 2018). These technologies contribute to reducing nursing staff fatigue ( Steege & Dykstra, 2016) and nurses’ stress, while increasing their ability to focus on patient care ( Ozan & Duman, 2020).

Tubaishat (2018) and Zhou *et al*. (2019) found that nurses age, sex, experience level and willingness to use the technology, are moderating variables of technology usage behavior, and predictors of perceived usability of EMR. On the other hand, the interface design of software functions plays a crucial role in technology implementation ( Tu *et al*., 2018).

In contrast, the lack of confidence by professionals, past experiences, heavy workloads, and the time needed to learn are barriers to use technology ( Wood *et al*., 2019). Additionally, nursing staff tend to use “informal workarounds” to deal with exceptional situations in administrative processes that have technology. These practices are a risk for patient safety ( van der Veen *et al*., 2020) and information reliability.

## Discussion

Technology in health services has benefits for streamlining processes, improving the quality of care, improving communication by supplying real-time information related to patient management and having data for decision making, process evaluation and results. The implementation success depends on the acceptance of technology.

Nurses have significant interaction with technology to monitor and manage patient care. Most studies look at the interaction of ICU nurses, but the technology requirement should extend to all health services, at all levels of complexity. Hence, an essential aspect in the implementation of technology is to enhance its acceptance ( Phillips, 2019).

IT and care management systems in nursing should allow effective planning of nursing workload. These tools should promote patient safety, quality of care, and staff satisfaction. This is achieved when technology allows staff to spend more time with the patient and less time doing administrative processes. Although the usefulness of technology for nursing practice and clinical decision making has been demonstrated, the study on the impact of these is incipient and there is no evidence on the best way to plan workload allocation in nursing ( Griffiths *et al*., 2020).

There are opportunities for research in this topic because the conclusions in the literature are contradictory. While some studies conclude that technology can increase staff productivity, empowerment, and improve nursing care outcomes, other’s show that technology can increase nursing staff stress and burnout. In addition, it is unknown how these technologies contribute to promote compassionate nursing care.

Further, the literature recognizes that studies analyzing the effect of technology use in nursing care is not free of bias ( Redley *et al*., 2020). In these cases, although observational practices do not allow us to see cause-effect relationships, it is feasible to think of methodologies that resolve these questions ( Griffiths *et al*., 2020).

Most of the software is limited because they don’t use real-time information. Instead, they work under the assumption that a sample is sufficient to generalize the needs of the patient population in the future. Furthermore, these solutions assume that once the contracted staffing level has been found, a fixed nurse-to-patient ratio and fixed number of hours will satisfy nursing care requirements ( Griffiths *et al*., 2020). The strongest assumption relies on average care requirement times and allocating the load assuming that individual variations can be accommodated ( Griffiths *et al*., 2020), ignoring the staff experience levels. Further, information systems should consider the scientific evidence showing that understaffing, low levels of skilled staffing, burnout, and high levels of intent to quit negatively affect clinical outcomes and increase turnover costs ( Simonetti *et al*., 2020). Simultaneously, user-friendly systems are needed to allow nurse managers to make better decisions quickly and efficiently without creating staff burnout and stress ( Harris *et al*., 2018).

The discipline of operational research techniques has proposed techniques to optimize staffing decisions using mathematical models or computer simulations, known as the “nurse rostering problem” and how to reassign staff to units with high demand. These algorithms have been able to prove that allocation based on averages may not be best in the face of variability in patient requirements and that this variability usually means more staffing to meet peak demand. However, how to calculate workload has not been discussed in depth ( Griffiths *et al*., 2020), and how to use IT to estimate and predict workload including all the physical, cognitive, organizational, and emotional dimensions still is a major unsolved question.

Finally, in this research we have not taken into consideration sex and gender differences in the results since the reviewed papers do not clearly conclude on how the associated factors and barriers that promote the technology acceptance and usability is different for different sex and gender.

### Limitations

Despite having a rigorous protocol for identifying scientific articles, there are few studies performed in non-critical services. The studies found have heterogeneous methodologies, which made it difficult to compare the evidence in the studies (see Table 1). Further, the literature review did not focus on studying the concept of innovation in nursing workload management as it is analysed in Rylee and Cavanagh (2022) for nursing practice.

## Conclusions

Based on the literature analysis, we conclude that IT has the potential to improve care management. There are several technological platforms that propose solutions to manage nurse workload and shift management. Further, the existing models use approaches based on expert opinion, benchmarking, nurse-patient relationship, patient prototyping, multifactorial indicators, and approaches based on task-based time requirements. These approaches differ importantly in how workload is assigned and the variables that are considered. It is equally important to analyze the technology and nursing workload in ICUs and non-critical units, but scientific evidence is more detailed in the former type of services.

The factors that promote the technology acceptance and usability of these models include the satisfaction for an increased time for direct care, a perception of improved patient safety, quality of care, and reduction of workload and staff burnout. The identified barriers for these technologies are the lack of knowledge of the technological tools, long times needed for their use, and low perceived ease of use. Thus, it is important to involve nursing professionals in the technological design process.

Our study reveals that most of the models and technologies are focused on intensive care units, and few studies for other types of care units. Also, all the considered studies are performed in developed economies, none of them are performed in economies in transition or developing economies. In addition, there is no scientific evidence to compare the technologies between each other and we found no scientific evidence showing improvements in care processes provided after using them. Further, with the proliferation of nursing workforce management systems, a review to evaluate efficacy is warranted. The main research gap that we identify is the lack of studies showing evidence of the impact of technology measured as the correlation between the nursing outcomes and staff well-being with the use of workload estimation and the strategies for planning workload allocation. One key contribution of this study is that the conclusions are obtained from the analysis of a multidisciplinary research team, including nurses with expertise in management and engineers with expertise in healthcare systems.

## Summary

### What is already known

• Evidence shows that the patient condition modifies the nursing workload. There is a relationship between the nursing workload, the morbidity, and mortality of hospitalized patients.
• Excessive nursing workload goes against the values of humanization of care, patient outcomes, patient safety, and quality of care.
• There is interest in creating technology to integrate scientific evidence and nursing expertise to reveal the relationship between nurse workload, burnout, and care quality.

### What this paper adds

• Current information technology for managing nursing workload partially contemplates evidence on nursing workload models, the predictors of nursing burnout and outcomes, and the theories of acceptance of technology. Existing technological platforms for nursing care management that propose solutions to manage nurse workload and shift management, each with different methodologies.
• Further, these technologies need to assess the nursing workload to capture the complexity or quality of care, and to recognize the concurrent, iterative, and interrelated nature of cognitive processes and activities in nursing. Evidence is needed to compare technologies and provide scientific evidence proving improvements in care *.*

## Data Availability

All data underlying the results are available as part of the article and no additional source data are required. OSF: PRISMA checklist for “Technological innovation for workload allocation in nursing care management: an integrative review”, https://doi.org/10.17605/OSF.IO/JXFYK ( Guerrero, 2022). PRISMA checklist is available under the terms of the Creative Commons Zero “No rights reserved” data waiver ( CC0 1.0 Universal (CC0 1.0) Public Domain Dedication).

## References

[ref1] Aiken LH Simonetti M Sloane DM : Hospital nurse staffing and patient outcomes in Chile: a multilevel cross-sectional study. *Lancet Glob. Health.* 2021;9(8):e1145–e1153. 10.1016/S2214-109X(21)00209-6 34224669

[ref2] Arango GL Peña B Vega Y : Relación de la asignación de personal de enfermería con indicadores de resultado de la calidad de la atención en unidades de cuidados intensivos de adultos. *Aquichan.* 2015;15(1):90–104. 10.5294/aqui.2015.15.1.9

[ref3] Aschan H Junttila K Fagerstrôm L : RAFAELA™ patient classification system as a tool for management. *Connecting Health and Humans.* IOS Press;2009; (pp.478–482). 19592889

[ref4] Assaye AM Wiechula R Schultz TJ : The impact of nurse staffing on patient and nurse workforce outcomes in acute care settings in low-and middle-income countries: a quantitative systematic review. *JBI Evidence Synthesis.* 2020. 10.11124/JBISRIR-D-19-00426 32881732

[ref5] Ball JE Bruyneel L Aiken LH : Post-operative mortality missed care and nurse staffing in nine countries: a cross-sectional study. *Int. J. Nurs. Stud.* 2018;78:10–15. 10.1016/j.ijnurstu.2017.08.004 28844649 PMC5826775

[ref6] Beaney P Hatfield R Hughes A : Creating digitally ready nurses in general practice. *Nurs. Manag.* 2019;26(3):27–35. 10.7748/nm.2019.e1840 31468839

[ref7] Berg GM LoCurto J Lippoldt D : Stages of adoption concern and technology acceptance in a critical care nursing unit. *J. Nurs. Adm.* 2017;47(9):441–447. 10.1097/NNA.0000000000000511 28834804

[ref8] Carlesi KC Padilha KG Toffoletto MC : Patient Safety Incidents and Nursing Workload. *Rev. Lat. Am. Enfermagem.* 2017;25:e2841. 10.1590/1518-8345.1280.2841 28403334 PMC5396482

[ref58] Chang LY Yu HH Chao YC : The Relationship Between Nursing Workload, Quality of Care, and Nursing Payment in Intensive Care Units. *The Journal of Nursing Research : JNR.* 2019;27(1):1–9. 10.1097/jnr.0000000000000265 PMC6369868 29613879

[ref9] Clark EL Poland M : *GRASP: Grace-Reynolds application and study of PETO.* Morganton, N.C: Kate B. Reynolds Health Care Trust to Grace Hospital;1976.

[ref10] Coster S Watkins M Norman IJ : What is the impact of professional nursing on patients’ outcomes globally? An overview of research evidence. *Int. J. Nurs. Stud.* 2018;78:76–83. 10.1016/j.ijnurstu.2017.10.009 29110907

[ref11] Crowe M Sheppard L Campbell A : Comparison of the effects of using the Crowe Critical Appraisal Tool versus informal appraisal in assessing health research: a randomised trial. *Int. J. Evid. Based Healthc.* 2011;9(4):444–449. 10.1111/j.1744-1609.2011.00237.x 22093394

[ref12] Dall’Ora C Ball J Reinius M : Burnout in nursing: a theoretical review. *Hum. Resour. Health.* 2020;18:1–17. 10.1186/s12960-020-00469-9 32503559 PMC7273381

[ref13] Oliveira AC de Garcia PC Nogueira L d S : Nursing workload and occurrence of adverse events in intensive care: A systematic review. *Rev. Esc. Enferm.* 2016;50:683–694. 10.1590/S0080-623420160000500020 27680056

[ref14] Farid M Purdy N Neumann WP : Using system dynamics modelling to show the effect of nurse workload on nurses’ health and quality of care. *Ergonomics.* 2020;63:952–964. 10.1080/00140139.2019.1690674 31696791

[ref15] Farzandipour M : Self-assessment of nursing informatics competencies in hospitals. *Online J. Nurs. Inform.* 2020;24(2).

[ref16] Giraldo L Schachner B Luna D : Exploring nurses’ perceptions and expectations toward a BCMA implementation using a mobile app and workstations as a change management strategy. *Nursing Informatics 2018.* IOS Press;2018; (pp.134–138). 29857405

[ref17] Griffiths P Saville C Ball J : Nursing workload, nurse staffing methodologies and tools: A systematic scoping review and discussion. *Int. J. Nurs. Stud.* 2020;103:103487. 10.1016/j.ijnurstu.2019.103487 31884330 PMC7086229

[ref60] Guerrero WJ : Technological innovation for workload allocation in nursing care management: An an integrative review. *PRISMA CHECKLIST.* 2022, December 26. 10.17605/OSF.IO/JXFYK PMC10904957 38434658

[ref18] Handayani PW Hidayanto AN Pinem AA : Hospital information system user acceptance factors: User group perspectives. *Inform. Health Soc. Care.* 2018;43(1):84–107. 10.1080/17538157.2016.1269109 28140717

[ref19] Harris DA Haskell J Cooper E : Estimating the association between burnout and electronic health record-related stress among advanced practice registered nurses. *Appl. Nurs. Res.* 2018;43:36–41. 10.1016/j.apnr.2018.06.014 30220361

[ref20] Harvey C Thompson S Otis E : Nurses’ views on workload, care rationing and work environments. *J. Nurs. Manag.* 2020;28(4):912–918. 10.1111/jonm.13019 32255223

[ref21] Higgins LW Shovel JA Bilderback AL : Hospital Nurses’ Work Activity in a Technology-Rich Environment. *J. Nurs. Care Qual.* 2017;32(3):208–217. 10.1097/NCQ.0000000000000237 28541263

[ref22] Holden RJ Asan O Wozniak EM : Nurses’ perceptions, acceptance, and use of a novel in-room pediatric ICU technology: testing an expanded technology acceptance model. *BMC Med. Inform. Decis. Mak.* 2016;16(1):110–145. 10.1186/s12911-016-0388-y 27846827 PMC5109818

[ref23] Hope J Griffiths P Schmidt PE : Impact of using data from electronic protocols in nursing performance management: A qualitative interview study. *J. Nurs. Manag.* 2019;27(8):1682–1690. 31482604 10.1111/jonm.12858 PMC6919414

[ref24] Italian Multicenter Group of ICU Research (GIRTI)., Iapichino, G: Time oriented score system (TOSS): A method for direct and quantitative assessment of nursing workload for ICU patients. *Intensive Care Med.* 1991;17:340–345. 10.1007/BF01716193 1744325

[ref25] Kang JH Kim CW Lee SY : Nurse-Perceived Patient Adverse Events depend on Nursing Workload. *Osong. Public Health Res. Perspect.* 2016;7:56–62. 10.1016/j.phrp.2015.10.015 26981344 PMC4776267

[ref26] Kim Y Kim HY Cho E : Association between the bed-to-nurse ratio and 30-day post-discharge mortality in patients undergoing surgery: a cross-sectional analysis using Korean administrative data. *BMC Nurs.* 2020;19:17. 10.1186/s12912-020-0410-7 32189999 PMC7076936

[ref27] Leary A Cook R Jones S : Mining routinely collected acute data to reveal non-linear relationships between nurse staffing levels and outcomes. *BMJ Open.* 2016;6(12):e011177. 10.1136/bmjopen-2016-011177 27986733 PMC5223722

[ref28] Magalhães AMMD Costa DGD Riboldi CDO : Association between workload of the nursing staff and patient safety outcomes. *Rev. Esc. Enferm. U.S.P.* 2017;51. 10.1590/s1980-220x2016021203255 29211232

[ref29] Maslach C Leiter MP : New insights into burnout and health care: Strategies for improving civility and alleviating burnout. *Med. Teach.* 2017;39(2):160–163. 10.1080/0142159X.2016.1248918 27841065

[ref30] Massarweh LJ Tidyman T Luu DH : Starting the Shift Out Right: The Electronic eAssignment Sheet Using Clinical Decision Support in a Quality Improvement Project. *Nurs. Econ.* 2017;35(4):194–200.

[ref31] Mau KA Fink S Hicks B : Advanced technology leads to earlier intervention for clinical deterioration on medical/surgical units. *Appl. Nurs. Res.* 2019;49:1–4. 10.1016/j.apnr.2019.07.001 31495412

[ref32] Miranda DR Nap R Rijk A de : Nursing activities score. *Crit. Care Med.* 2003;31(2):374–382. 10.1097/01.CCM.0000045567.78801.CC 12576939

[ref33] Moon WH : Development and evaluation of NRMIS (Nursing Resources Management Information System) for managing healthcare resources. *Technol. Health Care.* 2019;27(5):557–565. 10.3233/THC-191743 31156192

[ref34] Moreno-Monsiváis MG Moreno-Rodríguez C Interial-Guzmán MG : Missed nursing care in hospitalized patients. *Aquichan.* 2015;15(3):318–338. 10.5294/aqui.2015.15.3.2

[ref35] Ozan Y Duman M : Nurses’ Perceptions Regarding the Use of Technological Devices in Nursing Care Practices. *Int. J. Caring Sci.* 2020;13(2):901–908.

[ref36] Pedroso TG Pedrão LJ Perroca MG : Approaches to workload in psychiatric and mental health nursing. *Rev. Bras. Enferm.* 2020;73:e20190620. 10.1590/0034-7167-2019-0620 32696803

[ref37] Pekince H Aslan H : Determining the Work-Related Strain Levels of Nurses and Influencing Factors. *Int. J. Caring Sci.* 2020;13(1):135–142.

[ref38] Pendon RC Bitencourt JV d OV Haag FB : Evaluation of care technology applied to the nursing process in the light of best practices. *Rev Rene.* 2020;21:e44420. 10.15253/2175-6783.20202144420

[ref39] Phillips J : Complex Patient Care Technology. *AACN Adv. Crit. Care.* 2019;30(1):23–24. 10.4037/aacnacc2019730 30842070

[ref40] Redley B Douglas T Botti M : Methods used to examine technology in relation to the quality of nursing work in acute care: A systematic integrative review. *J. Clin. Nurs.* 2020;29(9-10):1477–1487. 10.1111/jocn.15213 32045059

[ref41] Redley B Richardson B Peel C : Co-development of “BRAIN-TRK”: Qualitative examination of acceptability, usability, and feasibility of an App to support nurses’ care for patients with behavioural and psychological symptoms of neurocognitive disorders in hospital. *J. Clin. Nurs.* 2019;28(15-16):2868–2879. 10.1111/jocn.14874 30938865

[ref42] Respicio A Moz M Pato MV : A computational application for multi-skill nurse staffing in hospital units. *BMC Med. Inform. Decis. Mak.* 2018;18(53):53. 10.1186/s12911-018-0638-2 29954378 PMC6025742

[ref43] Romero-Massa E Lorduy-Bolívar JP Pájaro-Melgar C : Relación entre la carga laboral de enfermería y la gravedad del paciente en unidades de cuidado intensivo de adultos. *Aquichan.* 2011;11(2):173–186. 10.5294/aqui.2011.11.2.4

[ref59] Rylee TL Cavanagh SJ : Innovation in Nursing Practice: A Scoping Review. *Adv. Nurs. Sci.* 2022. 10.1097/ANS.0000000000000464 36317833

[ref44] Sato K Yamashita K Goshima M : An Analysis of the Factor Model on the Workload of Nursing Staff Using a Hospital Management Tool. *Nursing Informatics.* 2016;58–62. 27332162

[ref45] Simonetti M Soto P Galiano A : Dotaciones, skillmix e indicadores laborales de enfermería en Hospitales Públicos chilenos. *Rev. Med. Chil.* 2020;148(10):1444–1451. 10.4067/S0034-98872020001001444 33844714

[ref46] Steege LM Dykstra JG : A macroergonomic perspective on fatigue and coping in the hospital nurse work system. *Appl. Ergon.* 2016;54:19–26. 10.1016/j.apergo.2015.11.006 26851460

[ref47] The Shelford Group: Safer nursing care tool implementation resource pack. 2014.

[ref49] Tu MH Chang P Lee YL : Avoiding obsolescence in mobile health: Experiences in designing a mobile support system for complicated documentation at long-term care facilities. *Comput. Inform. Nurs.* 2018;36(10):501–506. 10.1097/CIN.0000000000000460 30045129

[ref50] Tubaishat A : Perceived usefulness and perceived ease of use of electronic health records among nurses: Application of Technology Acceptance Model. *Inform. Health Soc. Care.* 2018;43(4):379–389. 10.1080/17538157.2017.1363761 28920708

[ref51] Tuominen O Lundgren-Laine H Teperi S : Comparing the two techniques for nursing staff rescheduling to streamline nurse managers’ daily work in finland. CIN. *Comput. Inform. Nurs.* 2020;38(3):148–156. 10.1097/CIN.0000000000000567 31652140

[ref52] Veen W van der Taxis K Wouters H : Factors associated with workarounds in barcode-assisted medication administration in hospitals. *J. Clin. Nurs.* 2020;29(13-14):2239–2250. 10.1111/jocn.15217 32043705 PMC7328795

[ref53] Vorakulpipat C Rattanalerdnusorn E Sirapaisan S : A mobile-based patient-centric passive system for guiding patients through the hospital workflow: design and development. *JMIR Mhealth Uhealth.* 2019;7(7):e14779. 10.2196/14779 31333195 PMC6681638

[ref54] Wood C Chaboyer W Carr P : How do nurses use early warning scoring systems to detect and act on patient deterioration to ensure patient safety? A scoping review. *Int. J. Nurs. Stud.* 2019;94:166–178. 10.1016/j.ijnurstu.2019.03.012 31002971

[ref55] Youn SH Son H Kim J : Trauma Versus Nontrauma Intensive Care Unit Nursing: A Workload Comparison. *J. Trauma Nurs.* 2020;27:346–350. 10.1097/JTN.0000000000000541 33156250

[ref56] Yu M-H Lee T-T Mills ME : The Effect of Barcode Technology Use on Pathology Specimen Labeling Errors. *AORN J.* 2019;109:183–191. 10.1002/aorn.12585 30694536

[ref57] Zhou LL Owusu-Marfo J Antwi HA : Assessment of the social influence and facilitating conditions that support nurses’ adoption of hospital electronic information management systems (HEIMS) in Ghana using the unified theory of acceptance and use of technology (UTAUT) model. *BMC Med. Inform. Decis. Mak.* 2019;19(1):1–9. 10.1186/s12911-019-0956-z 31752840 PMC6873399

