# Supplementary material for: Technological innovation for workload allocation in nursing care management: an integrative review
Source: F1000Res. 2024 Apr 26;12:104. Originally published 2023 Jan 27. [Version 3] doi: 10.12688/f1000research.125421.3 (PMC10904957; doi:10.12688/f1000research.125421.3)
Supplement: Peer review history

# Reviewer report: Reviewer response for version 3

**DOI:** 10.5256/f1000research.164355.r269656

**Published:** 2024-05-23

**Authors:** Lorena Chaparro-Diaz

**Affiliations:** 1 Universidad Nacional de Colombia, Bogotá, Bogota, Colombia

The authors have incorporated the suggested adjustments in improvements that are susceptible to do so. However, the discussion is still compared with references that are not recent from the last 2 years, this is important because the study is precisely a review article and by the time of indexing would be expected to discuss the results with new literature, I did not find a justification in the text for not incorporating the adjustment.

Is the review written in accessible language?

Yes

Are all factual statements correct and adequately supported by citations?

Yes

Are the conclusions drawn appropriate in the context of the current research literature?

Yes

Is the topic of the review discussed comprehensively in the context of the current literature?

Yes

Reviewer Expertise:

Chronic illness ; Research ; Caregiver ; Care Policy ; Nursing ; Qualitative research ; Nursing care ; Education ; Health Policy

I confirm that I have read this submission and believe that I have an appropriate level of expertise to confirm that it is of an acceptable scientific standard.

# Reviewer report: Reviewer response for version 3

**DOI:** 10.5256/f1000research.164355.r269657

**Published:** 2024-05-13

**Authors:** Claudio-Alberto Rodríguez-Suárez

**Affiliations:** 1 University of Las Palmas de Gran Canaria, Las Palmas, Spain

Dear Authors,

 In my opinion some methodological aspects in the manuscript should be improved.

 Since it is an integrative review, you should include scientific literature and gray literature. In the inclusion and exclusion criteria section, the study designs included and excluded should be detailed.

 Figure 1 (flowchart) includes the search results, which in a review is the first result; therefore, Figure 1 should be transferred to the results section and the process of identification and screening of records and inclusion of studies should be described in results.

 I believe that the rest of the manuscript is correct after the authors' modifications to the reviewers' comments.

Is the review written in accessible language?

Partly

Are all factual statements correct and adequately supported by citations?

Partly

Are the conclusions drawn appropriate in the context of the current research literature?

Yes

Is the topic of the review discussed comprehensively in the context of the current literature?

Partly

Reviewer Expertise:

Nursing

I confirm that I have read this submission and believe that I have an appropriate level of expertise to confirm that it is of an acceptable scientific standard, however I have significant reservations, as outlined above.

# Reviewer report: Reviewer response for version 2

**DOI:** 10.5256/f1000research.156435.r240899

**Published:** 2024-02-16

**Authors:** Nancy Purdy

**Affiliations:** 1 Nursing, Toronto Metropolitan University, Toronto, Ontario, Canada

The authors conducted an integrated review of the literature to analyze technology-based tools for their use and impact on nursing workload models, burnout predictors and outcomes as well as factors that influence technology acceptance by nursing professionals. PRSIMA guidelines were used to inform the methods for the literature review. The flowchart of the screening process was accurately and clearly presented. All selected papers were summarized in Table 1 and elaborated upon in the results section. The discussion that followed included a summary of key findings to address the study aims. The conclusions aligned well with the results and discussion sections.

 While the overall structure of the paper is adequate, the paper could be improved significantly with a few revisions.

1. The main issue is the need to clearly and consistently articulate the study aims and align these aims with the study title. In the abstract, the aims include an analysis of nursing workload models, predictors of nursing burnout and outcomes and the acceptance of new technologies for nursing care management. Each of these aims seem divergent and each has a large body of literature. Considering the title of the paper, I think something is missing in the explanation of the study aims. I have assumed that aim is in fact the one that I have noted in the first paragraph i.e. the use and impact of technological innovations or tools for nursing care management which includes those used for nursing workload models, the impact of technological innovations on predictors and outcomes of burnout and overall nursing acceptance of these technological tools. In effect, I am assuming that the authors are intending to report on the integration of all of these concepts – technology and nursing workload, burnout and user acceptance. Therefore, the research questions on page 3 need to be more explicit. For RQ1, a suggested revision would be,  “ What methods are used to calculate nursing workload?”. The concept of nursing workload should also be defined i.e. are you referring to nursing workload measurement? RQ2 is unclear and seems to overlap with RQ1. RQ3 is stated clearly. Any changes made to improve clarity should be reflected in the abstract, methods and remaining sections of the paper.
2. The search strategy was described but the inclusion and exclusion criteria for the selection of papers should be elaborated upon with sufficient detail that someone else could replicate the study. The authors stated “Articles related with technology that didn’t analyze the impact of nursing workload or didn’t present technologies were excluded.” This is helpful and lead me to conclude that articles needed to address the integration of technology on nursing workload, burnout and nursing acceptance. This needs to be stated more explicitly.
3. The organization and content of Table 1 needs to be revised to reflect the study aims. Some papers only address one of the concepts of interest but not necessarily the integration of technology and nursing workload, burnout and acceptance. I think that this might be due to the content provided in the ‘outcomes’ column. Perhaps the content selected for this column could be revised to reflect this aim more clearly. Also, the order in which papers are presented does not seem to follow any rules (not listed in chronological or alphabetical order) nor listed by the concept of interest. For example, the column header for ‘Predictors of nursing burnout’ lists a paper by Hope *et al. *but the paper summary does not mention burnout. On the next page (p.7), the column header is ‘Nursing Workload Models’ but there is a paper by Dall’Ora on burnout. It would be useful to have clear subheadings the reflect the order of the concepts as presented in the aim statement. Also, it is not clear why some papers were selected as there is no mention of technology in the summaries. Perhaps more clarity in the aim statement, inclusion/exclusion criteria and revisions to the ‘outcomes’ column would help to justify the papers selected and reported in this table.
4. The concept map is very useful and helps the reader to understand the results of the content analysis. I have two suggestions for your consideration.
  1. In the middle column, I would consider nursing workload models and information technologies to be the main elements of nursing care management. I think that burnout predictors and outcomes would better fall within the third column as they are not a type of management tool. Similarly, I think technology acceptance would better fit under the third column as well as it is not an element of nursing care management but instead is a factor related to technologies.
  2. Suggest adding a 4 ^th^ column to list the authors for the papers where related evidence for the theme was found. This will help the reader with understanding the results and discussion sections as well.
5. Other recommended edits – There are places that require further editing to improve the sentence structure but I have not listed them here (available if requested). The abstract requires the most work  (primarily the background and methods sections) as there are duplicate phrases or unclear sentences. Also, human factors is mentioned in the background section but is not discussed anywhere in the paper.

Overall, I think this is a useful research report that adds to our understanding of the impact of technology on nursing workload. I think the proposed revisions will add to the uptake and use of this review.

Is the review written in accessible language?

Yes

Are all factual statements correct and adequately supported by citations?

Yes

Are the conclusions drawn appropriate in the context of the current research literature?

Yes

Is the topic of the review discussed comprehensively in the context of the current literature?

Yes

Reviewer Expertise:

nursing work environments, leadership, nursing workload measurement, program evaluation, quantitative research

I confirm that I have read this submission and believe that I have an appropriate level of expertise to confirm that it is of an acceptable scientific standard, however I have significant reservations, as outlined above.

# Reviewer report: Reviewer response for version 2

**DOI:** 10.5256/f1000research.156435.r231640

**Published:** 2024-02-14

**Authors:** Claudio-Alberto Rodríguez-Suárez

**Affiliations:** 1 University of Las Palmas de Gran Canaria, Las Palmas, Spain

Dear authors, thank you very much for the opportunity to review this manuscript. This systematic review addresses an interesting topic on nurses' workloads. Since this is a systematic review study, the following comments are based on the PRISMA guidelines.

 As general comments, a quality criterion for a systematic review is the registration of the review protocol (for example in PROSPERO). The registration number should be provided in the method and (according to PRISMA also in the abstract).

 Abstract: is structured and informative.In the methods section it is possible to improve the information on the following aspects: Specify the inclusion and exclusion criteria. "Cinahl" is badly written. In the summary you duplicate the explanation of the Critical Appraisal Tool version 1.4 (CCAT) acronym. The acronyms ICUs and PRISMA are not explained.

 Keywords: For proper indexing of the article once it is published, it is appropriate to use keyword descriptor terms (MesH). "Nursing Care Management" is not a MesH. ("Nursing Care" is a MesH; there are several terms that include Management that can also be used. Regarding the two terms "Personnel Staffing", "Scheduling Information Systems": there is a single descriptor that can include both "Personnel Staffing and Scheduling Information Systems".

 Introduction: The introduction is very sparse; content on Technological innovation and nurses workload could be included.

 Methods: It is not understood that you have established among the inclusion criteria searches until the year 2020. Three years have passed since the date of the searches and in that period new studies of interest could have been published. It is a quality criterion to update the searches when at least one year has passed since they were performed if the manuscript has not been published in that period.

 Results: The results should be limited to the extraction of data from the studies without interpretation to be made in the discussion. The quality criteria of the studies should be explained in detail in the methods section. When looking at the results in Table 1, it is not possible to interpret the criterion in the last column: Excellent, some limitations...

 Characteristics of the studies: in the description of the number of studies included, you must unify criteria to express them in numbers or in letters (example: four vs. 4).

 The acronym ICUs is explained in results (heading: Predictors of nursing burnout and outcomes; last paragraph of this section), but the acronym has been used previously in the same paragraph. Idem EMRs acronym.

 Discussion: The discussion is poor, only citing 5 studies. As I have pointed out in the previous comment, you must transfer content from the results to the discussion. The discussion should follow the structure established in the results.

Is the review written in accessible language?

Partly

Are all factual statements correct and adequately supported by citations?

Partly

Are the conclusions drawn appropriate in the context of the current research literature?

Yes

Is the topic of the review discussed comprehensively in the context of the current literature?

Partly

Reviewer Expertise:

Nursing

I confirm that I have read this submission and believe that I have an appropriate level of expertise to state that I do not consider it to be of an acceptable scientific standard, for reasons outlined above.

# Reviewer report: Reviewer response for version 1

**DOI:** 10.5256/f1000research.137724.r178450

**Published:** 2023-08-10

**Authors:** Angela María Salazar Maya

**Affiliations:** 1 Universidad de Antioquia, Medellín, Colombia

It is a relevant issue for nursing as a discipline and profession, since evidence is required on how the workload influences the results of the patients cared for.

 The search was only carried out until the year 2020, it would be pertinent to extend the search until at least 2022 or the first half of 2023 so that the integrative review is more up-to-date.

 The paper presents the 4 categories that emerge from the analysis: workload models, burnout predictors, and outcomes; information technology and the meaning of technology for management; technology acceptance. develop each category.

 I consider the discussion and conclusions pertinent.

 In a brief search carried out in PubMed, I find articles on the subject.

Is the review written in accessible language?

Yes

Are all factual statements correct and adequately supported by citations?

Yes

Are the conclusions drawn appropriate in the context of the current research literature?

Yes

Is the topic of the review discussed comprehensively in the context of the current literature?

Yes

Reviewer Expertise:

Epistemology of nursing, perioperative care, family caregivers, qualitative research

I confirm that I have read this submission and believe that I have an appropriate level of expertise to confirm that it is of an acceptable scientific standard.

## References

[1] : A systematic review study on the factors affecting shortage of nursing workforce in the hospitals. *Nurs Open* .2023;10(3) : 10.1002/nop2.1434 1247-1257 10.1002/nop2.1434 36303066 PMC9912424

[2] : Nurse staffing levels and patient outcomes: A systematic review of longitudinal studies. *Int J Nurs Stud* .2022;134: 10.1016/j.ijnurstu.2022.104311 104311 10.1016/j.ijnurstu.2022.104311 35780608

# Reviewer report: Reviewer response for version 1

**DOI:** 10.5256/f1000research.137724.r178447

**Published:** 2023-07-12

**Authors:** Lorena Chaparro-Diaz

**Affiliations:** 1 Universidad Nacional de Colombia, Bogotá, Bogota, Colombia

**Abstract**

 This article is an integrated review of the scientific literature produced in 3 languages (Spanish, English, and Portuguese) that sought to analyze nursing workload models, predictors of overload and outcomes, and new technologies in nursing care management. The authors used as a methodological framework PRISMA elements that allowed them to analyze 35 articles in full text, was conducted by an interdisciplinary team integrating nurses and engineers, to understand the phenomenon adequately. The results showed the potential of technology in care management, but there is a lack of systems and validated technology that would allow usable nursing workload models. Barriers to the use of technology that could be associated with work overload were identified; however, there is more evidence of the effects of burnout on patient outcomes and adverse events. Their findings are located more in critical care settings.

**Strengths**

 Among the strengths of the review is that it contributes to a need for summative evidence on aspects of nursing care management that had not been identified as influential, such as nursing workload. It recognizes current elements such as the use of technologies for nursing care and concludes that nurses need to work collaboratively with software programmers for the development of new technologies.

 Another important aspect is the identification of at least some existing systems, especially in critical care, which could be taken up again to compare their use in future studies. 

 The review also validates with more evidence that excessive nursing workload has negative impacts on the health of the people cared for, which is a useful manuscript for nurse managers to justify institutional changes.  

 In nursing, real-time patient assessment has always been key, which would allow the development of schemes with technology; however, it highlights that causality is not the only way of nursing care, and qualitative aspects of care that are performed with humanization and compassionate care should be contemplated; this gives a comprehensive paradigm to the reader of the nursing role.

**Weaknesses**

 The article could improve its presentation of aspects that may be justifiable from the methodology used, such as answering the question: Why did not they use the new PRISMA 2020 scheme? For an integrative review, it should not necessarily be used, but in the methodology, this methodology is used, but it is not mentioned what is or is not used and why. It is not possible to identify the elements suggested in the PRISMA 2020 parameters, regarding aspects such as the date on which each resource was last consulted. It is not clear whether the authors were involved in the screening stages in the selection or only in the phase of reading abstracts. 

 Table 1 has very valuable information for readers; however, one could be more assertive in the presentation by having more heterogeneity in the presentation of the results in the Data Collection Methods and Results column; it may be that having been elaborated by different authors of the article or supporting persons, the presentation of the information varies too much. Now, it may have been the effect of the limitation they presented in the manuscript that it was too heterogeneous, which they could specify there and cite as an example in Table 1, where this can be seen.

**Improvements to the article** 

 The manuscript can also be improved with aspects such as updated references. In the introduction, they have a citation from 2011 (12 years’ difference), on a topic that could have more recent references. 

 The discussion could include aspects of recently published discussions (last two years until 2023) since they were limited to the same studies of the review. 

 I suggest being more precise in stating the research gaps identified, which may guide readers on topics of research interest, lacking this specification. 

 Finally, from origin of the article, I suggest reviewing if the word "innovation" is pertinent in the article, since it relates it to the topic of technologies that is in the research questions; however, not necessarily when talking about technology it is associated to an innovation, a technology can be an artifact or a technique; while innovation is to create or modify a product, but it is not what was sought in the review nor was it what was found in the studies.

**Final conclusion of the concept**

 The manuscript is very good in its content, richness, rigorous work, concise, and useful for care management. It can be improved with precisions and updates typical of literature reviews. I congratulate the authors for the phenomenon addressed and the possibility of having this type of review.

Is the review written in accessible language?

Yes

Are all factual statements correct and adequately supported by citations?

Yes

Are the conclusions drawn appropriate in the context of the current research literature?

Yes

Is the topic of the review discussed comprehensively in the context of the current literature?

Yes

Reviewer Expertise:

Chronic illness ; Research ; Caregiver ; Care Policy ; Nursing ; Qualitative research ; Nursing care ; Education ; Health Policy

I confirm that I have read this submission and believe that I have an appropriate level of expertise to confirm that it is of an acceptable scientific standard, however I have significant reservations, as outlined above.

# Response

**Published:** 2023-09-22

**Authors:** William Guerrero

**Affiliations:** Universidad de La Sabana, Colombia

We thank the reviewer for the valuable comments made on version 1 of the manuscript. We answer the reviewers’ comments as follows:

- Why did not they use the new PRISMA 2020 scheme?  For an integrative review, it should not necessarily be used, but in the methodology, this methodology is used, but it is not mentioned what is or is not used and why. It is not possible to identify the elements suggested in the PRISMA 2020 parameters, regarding aspects such as the date on which each resource was last consulted. It is not clear whether the authors were involved in the screening stages in the selection or only in the phase of reading abstracts.

Answer: The first paragraph of the Methods section provides the details of the methodology used that corresponds to the PRISMA 2020 guidelines as suggested by the reviewer. The corresponding paragraph is as follows:

An integrative literature review was performed using the Preferred Reporting Items for Systematic Reviews and Meta-Analyses (PRISMA) 2020 guidelines. Studies were retrieved from SCOPUS, Scielo, PUBMED, and CINAHL in January 2021. The search strategy was: ("analytics" OR "operations research" OR digital* OR "IT" OR technolog*) AND ("nursing workload" OR "nurse-patient ratio" OR "nursing workforce planning" OR "nursing algorithm" OR "nursing personnel staffing and scheduling" OR "nursing staff")). The authors were involved in the identification, screening, and analysis stages. Inclusion criteria considered studies published in full text in English, Spanish or Portuguese classified as scientific articles or reviews, in the areas of nursing, medicine, social sciences, engineering, published between January 2016 and December 2020. This date range was considered to focus on the recent technologies. Articles related with technology that didn’t analyse the impact on nursing workload or didn’t present technologies were excluded. For all research, this must include a final section including details of ethical approval, informed consent and, where relevant registration.

  

- Table 1 has very valuable information for readers; however, one could be more assertive in the presentation by having more heterogeneity in the presentation of the results in the Data Collection Methods and Results column; it may be that having been elaborated by different authors of the article or supporting persons, the presentation of the information varies too much. Now, it may have been the effect of the limitation they presented in the manuscript that it was too heterogeneous, which they could specify there and cite as an example in Table 1, where this can be seen.

Answer: we have improved table 1. We included more information about references Redley et al. (2019), Hope et al. (2019), Kang, Kim, Lee (2016), Sato et al. (2016), Vorakulpipat et al. (2019), Zhou et al. (2019), Higgins et al. (2017), Tu et al. (2018), Van der Veen et al. (2020), Handayani et al. (2018), De Oliveira, Garcia, Nogueira (2016). The records on this new table are more homogeneous. Nevertheless, we still acknowledge the study limitation about the heterogeneous methodologies in the retrieved studies and we cite the example in the limitations.

  

- In the introduction, they have a citation from 2011 (12 years’ difference), on a topic that could have more recent references.

Answer: To update the references, we have included the following citation: Chang, L. Y., Yu, H. H., & Chao, Y. C. (2019). The Relationship Between Nursing Workload, Quality of Care, and Nursing Payment in Intensive Care Units. The journal of nursing research : JNR, 27(1), 1–9. https://doi.org/10.1097/jnr.0000000000000265

  

- The discussion could include aspects of recently published discussions (last two years until 2023) since they were limited to the same studies of the review.

Answer: It is true that our discussion section is built upon the studies that were retrieved by the proposed systematic review. In fact, we aimed to draw conclusions appropriate in the context of the current research literature and minimize bias and lack of rigor (Hopia et al., 2016). In fact, we aimed to avoid common pitfalls in integrative literature reviews that are described by Toronto and Remington (2020) the document entitled: “A Step-by-Step Guide to Conducting an Integrative Review”. The pitfalls are:

-
  - Drawing conclusions or formulating implications that cannot be supported by the literature,
  - Inserting new information/citations in the conclusion section of the review

Hopia, H., Latvala, E., & Liimatainen, L. (2016). Reviewing the methodology of an integrative review. *Scandinavian journal of caring sciences*, *30*(4), 662-669.

 Toronto, C. E., & Remington, R. (Eds.). (2020). *A step-by-step guide to conducting an integrative review* (pp. 1-9). Cham: Springer International Publishing.

  

- I suggest being more precise in stating the research gaps identified, which may guide readers on topics of research interest, lacking this specification.

Answer: We conclude that “The main research gap that we identify is the lack of studies showing evidence of the impact of technology measured as the correlation between the nursing outcomes and staff well-being with the use of workload estimation and the strategies for planning workload allocation”. This conclusion is presented in the final section of the paper. 

  

- I suggest reviewing if the word "innovation" is pertinent in the article, since it relates it to the topic of technologies that is in the research questions; however, not necessarily when talking about technology it is associated to an innovation, a technology can be an artifact or a technique; while innovation is to create or modify a product, but it is not what was sought in the review nor was it what was found in the studies.

Answer: We declare as a limitation of the study that the literature review did not focus on studying the concept of innovation in nursing practice or workload management as in Rylee and Cavanagh (2022) for nursing practice.

 We thank the reviewer for the valuable comments made on version 1 of the manuscript. We answer the reviewers’ comments as follows:

- Why did not they use the new PRISMA 2020 scheme?  For an integrative review, it should not necessarily be used, but in the methodology, this methodology is used, but it is not mentioned what is or is not used and why. It is not possible to identify the elements suggested in the PRISMA 2020 parameters, regarding aspects such as the date on which each resource was last consulted. It is not clear whether the authors were involved in the screening stages in the selection or only in the phase of reading abstracts.

Answer: The first paragraph of the Methods section provides the details of the methodology used that corresponds to the PRISMA 2020 guidelines as suggested by the reviewer. The corresponding paragraph is as follows:

An integrative literature review was performed using the Preferred Reporting Items for Systematic Reviews and Meta-Analyses (PRISMA) 2020 guidelines. Studies were retrieved from SCOPUS, Scielo, PUBMED, and CINAHL in January 2021. The search strategy was: ("analytics" OR "operations research" OR digital* OR "IT" OR technolog*) AND ("nursing workload" OR "nurse-patient ratio" OR "nursing workforce planning" OR "nursing algorithm" OR "nursing personnel staffing and scheduling" OR "nursing staff")). The authors were involved in the identification, screening, and analysis stages. Inclusion criteria considered studies published in full text in English, Spanish or Portuguese classified as scientific articles or reviews, in the areas of nursing, medicine, social sciences, engineering, published between January 2016 and December 2020. This date range was considered to focus on the recent technologies. Articles related with technology that didn’t analyse the impact on nursing workload or didn’t present technologies were excluded. For all research, this must include a final section including details of ethical approval, informed consent and, where relevant registration.

  

- Table 1 has very valuable information for readers; however, one could be more assertive in the presentation by having more heterogeneity in the presentation of the results in the Data Collection Methods and Results column; it may be that having been elaborated by different authors of the article or supporting persons, the presentation of the information varies too much. Now, it may have been the effect of the limitation they presented in the manuscript that it was too heterogeneous, which they could specify there and cite as an example in Table 1, where this can be seen.

Answer: we have improved table 1. We included more information about references Redley et al. (2019), Hope et al. (2019), Kang, Kim, Lee (2016), Sato et al. (2016), Vorakulpipat et al. (2019), Zhou et al. (2019), Higgins et al. (2017), Tu et al. (2018), Van der Veen et al. (2020), Handayani et al. (2018), De Oliveira, Garcia, Nogueira (2016). The records on this new table are more homogeneous. Nevertheless, we still acknowledge the study limitation about the heterogeneous methodologies in the retrieved studies and we cite the example in the limitations.

  

- In the introduction, they have a citation from 2011 (12 years’ difference), on a topic that could have more recent references.

Answer: To update the references, we have included the following citation: Chang, L. Y., Yu, H. H., & Chao, Y. C. (2019). The Relationship Between Nursing Workload, Quality of Care, and Nursing Payment in Intensive Care Units. The journal of nursing research : JNR, 27(1), 1–9. https://doi.org/10.1097/jnr.0000000000000265

  

- The discussion could include aspects of recently published discussions (last two years until 2023) since they were limited to the same studies of the review.

Answer: It is true that our discussion section is built upon the studies that were retrieved by the proposed systematic review. In fact, we aimed to draw conclusions appropriate in the context of the current research literature and minimize bias and lack of rigor (Hopia et al., 2016). In fact, we aimed to avoid common pitfalls in integrative literature reviews that are described by Toronto and Remington (2020) the document entitled: “A Step-by-Step Guide to Conducting an Integrative Review”. The pitfalls are:

-
  - Drawing conclusions or formulating implications that cannot be supported by the literature,
  - Inserting new information/citations in the conclusion section of the review

Hopia, H., Latvala, E., & Liimatainen, L. (2016). Reviewing the methodology of an integrative review. *Scandinavian journal of caring sciences*, *30*(4), 662-669.

 Toronto, C. E., & Remington, R. (Eds.). (2020). *A step-by-step guide to conducting an integrative review* (pp. 1-9). Cham: Springer International Publishing.

  

- I suggest being more precise in stating the research gaps identified, which may guide readers on topics of research interest, lacking this specification.

Answer: We conclude that “The main research gap that we identify is the lack of studies showing evidence of the impact of technology measured as the correlation between the nursing outcomes and staff well-being with the use of workload estimation and the strategies for planning workload allocation”. This conclusion is presented in the final section of the paper. 

  

- I suggest reviewing if the word "innovation" is pertinent in the article, since it relates it to the topic of technologies that is in the research questions; however, not necessarily when talking about technology it is associated to an innovation, a technology can be an artifact or a technique; while innovation is to create or modify a product, but it is not what was sought in the review nor was it what was found in the studies.

Answer: We declare as a limitation of the study that the literature review did not focus on studying the concept of innovation in nursing practice or workload management as in Rylee and Cavanagh (2022) for nursing practice.
